# Supplementary material for: The Small RNA Universe of Capitella teleta
Source: Front Mol Biosci. 2022 Feb 25;9:802814. doi: 10.3389/fmolb.2022.802814 (PMC8915122; doi:10.3389/fmolb.2022.802814)
Supplement: Supplementary file 1 [file DataSheet1.ZIP › Supplement/confident/CAPTEscaffold_94_8186.pdf]

Provisional ID : CAPTEscaffold_94_8186
Score total : 272.3
Score for star read(s) : 3.9
Score for read counts : 264.7
Score for mfe : 2.7
Score for randfold : 1.6
Score for cons. seed : -0.6
Total read count : 531
Mature read count : 508
Loop read count : 0
Star read count : 23

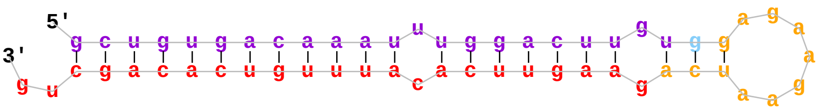

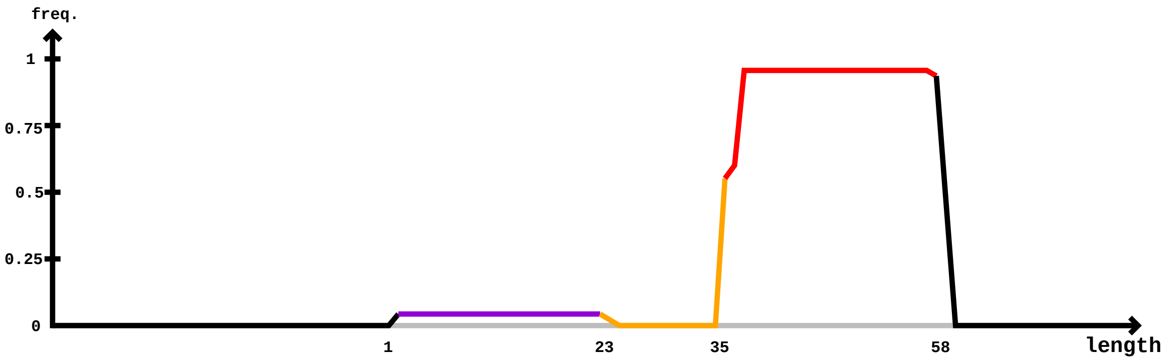


```
                                   Star                              Mature
5'- cauguuguugucaacguuauuacuaugcaugucagcugugacaaauuuggacuuguggagaagaaucagaaguucacauuugucacagcugacaugcauaguaauaacguu  -3'  obs
    cauguuguugucaacguuauuacuaugcaugucagcugugacaaauuuggacuuguggagaagaaucagaaguucacauuugucacagcugacaugcauaguaauaacguu       exp
    ............((((((((((((((((((((((((((((((((((.(((((((.(((.......))).))))))).))))))))))))))))))))))))))))))))))   reads   mm   sample
    ..................................gcugugacaaauuuggacuugu.......................................................   21      0    seq
    ..................................gcugugacaaauuuggacuugug......................................................   2       0    seq
    ....................................................................gaaguucacauuugucGcagcu.....................   3       1    seq
    ....................................................................gaaguucacauuugucacagcu.....................   2       0    seq
    ....................................................................gaGguucacauuugucacagcug....................   1       1    seq
    ....................................................................gGaguucacauuugucacagcug....................   11      1    seq
    ....................................................................gaaguucacauuugucacagcug....................   17      0    seq
    ....................................................................gaaguucacauuugucGcagcug....................   259     1    seq
    .....................................................................aaguucacauuugucacagcu.....................   1       0    seq
    .....................................................................aaguucacauuugucGcagcu.....................   4       1    seq
    .....................................................................aaguucacauuugucacagcCg....................   1       1    seq
    .....................................................................aaguucacauuugucacagcug....................   8       0    seq
    .....................................................................aaguucacauuugucGcagcug....................   11      1    seq
    .....................................................................aaguucacauuugucacagcuga...................   1       0    seq
    ......................................................................aguucacauuugucacagcu.....................   1       0    seq
    ......................................................................aguucacauuugucacGgcug....................   6       1    seq
    ......................................................................aguucacauuugucacagcug....................   149     0    seq
    ......................................................................aguucacauuugucGcagcug....................   28      1    seq
    ......................................................................aguucacauGgucacagcug....................   1       1    seq
    ......................................................................aguucacauuugucacagcugU...................   4       1    seq
```
